# Using Routinely Collected Clinical Assessments in Mental Health Services: The Resident Assessment Instrument–Mental Health

**DOI:** 10.1177/070674371405900708

**Published:** 2014-07

**Authors:** Chris Perlman, Lynn Martin, John Hirdes

**Affiliations:** Waterloo, Ontario; Thunder Bay, Ontario; Waterloo, Ontario

Dear Editor:

Dr Urbanoski and colleagues^1^ examined the use of the Resident Assessment Instrument–Mental Health (RAI-MH) for specialized inpatient mental health services. While the article underscores the importance of a comprehensive approach to implementation (for example, training or information technology infrastructure), much of the critique appears to reflect a lack of understanding of the design and applications of the RAI-MH. Urbanoski et al imply that the RAI-MH system was developed outside of real-world contexts when, in fact, front-line clinicians were engaged in all aspects of the development and refinement of the instrument and its applications. Numerous studies since the development work were based on data collected within routine clinical practice, including research on the Cognitive Performance Scale,^2^ Clinical Assessment Protocols,^3^,^4^ and quality indicators.^5^

The suggestion that most RAI-MH scales are “irrelevant for most patients”^1^^, p 692^ is particularly surprising and misguided. The authors incorrectly identified several scales as outcome measures, or had flawed operationalizations of specific scales. For example, the embedded CAGE (Cut down, Annoyed, Guilty, and Eye-opener) index was evaluated as an outcome measure when it was intended only to be used as a screener for substance abuse. The authors failed to consider the 90-day, look-back period for the RAI-MH items used to populate the CAGE (that is, there may have been overlap between time 1 and 2 observations). Further, conclusions that the RAI-MH lacks indicators of addiction severity are misleading, given that it includes numerous items related to substance and alcohol use, gambling, mental state, involvement with the criminal justice system, and vocational and interpersonal functioning. These measures provide ample opportunity to derive meaningful indices of addiction severity.

Urbanoski et al^1^ also appear to have incorrectly calculated scale values in their study, which makes their conclusions about the use of these scales among specialized populations questionable. A range of 0 to 8 was reported for the Positive Symptom Scale (PSS), though this scale should range from 0 to 12. We analyzed RAI-MH data provided by the Canadian Institute for Health Information for 276 055 people with and without schizophrenia in 75 hospitals across Ontario between 2005 and 2012. The mean PSS was 1.20 (SD 2.22) for people without schizophrenia, and 4.15 (SD 3.25) among people with schizophrenia or other psychotic disorders. For people with schizophrenia, an effect size of 1.32 was found for change in the PSS between admission and discharge assessments. These findings provide clear evidence in support of the PSS.

Urbanoski et al^1^ conclude that the difficulties experienced by a single organization’s implementation of an assessment system cannot be attributed to “either to the assessment platform or to issues of staff motivation and compliance.”^1^^, p 693^ Real-world evidence from 74 other hospitals would appear to contradict Urbanoski et al’s report. It is concerning that staff interviewed in this study identified little value in an assessment that includes items paramount to mental health care, including harm to self and others, social and vocational functioning, and traumatic life events, among others previously mentioned. Perhaps the implementation of innovative decision support applications for the RAI-MH in shared clinical decision-making contexts may enhance applications of this system.
