# Supplementary material for: Using Routinely Collected Clinical Assessments in Mental Health Services: The Resident Assessment Instrument–Mental Health
Source: Can J Psychiatry. 2014 Jul;59(7):399–400. doi: 10.1177/070674371405900708 (PMC4086312; doi:10.1177/070674371405900708)
Supplement: Related texts

# Response: Re: Using Routinely Collected Clinical Assessments in Mental Health Services: The Resident Assessment Instrument–Mental Health

**Published:** 2014-07

**Authors:** Karen Urbanoski, Benoit H Mulsant, Peggie Willett, Brian Rush

**Affiliations:** Toronto, Ontario

Dear Editor:

We appreciate the attention that Dr Perlman et al^1^ have brought to our work^2^ with their letter, and are happy to take this opportunity to both clarify some of our original interpretations and to respectfully disagree on certain points they raise.

First, we did not intend to imply that the Resident Assessment Instrument–Mental Health (RAI-MH) system was developed outside of clinical contexts. We used the phrase real-world to describe our own focus on the implementation and clinical use of the system at one facility, not to contrast it favourably with the work done by the developers.

Second, Dr Perlman et al^1^ challenge our argument that most RAI-MH scales are irrelevant for most patients. While the suite of scales captures the psychopathology of most patients, only a few of the scales will apply to a given patient. This, in turn, results in skewed distributions and very low mean scores on most of the scales when they are used to characterize the patient population. This property of measures based on symptom assessments, and the resulting difficulties in scaling patient-level improvements, has been echoed elsewhere.^3^ We take responsibility if we were not sufficiently clear on this point.

Third, Perlman et al^1^ suggest that we incorrectly identified scales as outcomes, giving the CAGE-AID (Cut down, Annoyed, Guilty, and Eye-opener–Questions Adapted to Include Drug use) as an example. We did not evaluate the CAGE-AID as an outcome; see Tables 3 to 5 in the article for the list of scales that were evaluated as outcomes.^2^ The confusion perhaps arises because we refer to the CAGE-AID when noting the lack of addiction-focused outcomes.^2^^, p 694^ Similarly, we did not fail to note the 90-day window on this measure; however, the potential overlap of recall windows would scarcely explain why only 12% of all patients, and only 36% of patients with a chart diagnosis of a substance use disorder, screened positive on the CAGE-AID.

Fourth, we stand by our argument that the RAI-MH system is not particularly well-suited to assessment or outcome monitoring in addictions service settings. The RAI-MH does capture information on the recency of substance use and selected domains of interest (listed in Perlman et al^1^); however, it does not capture, for example, volume of use, route of administration, motivation for treatment, or for changing substance use, problem substances, family history, substance use within the client’s social network, sexual health, or most of the myriad of illegal behaviours associated with substance use and addiction. Moreover, the RAI-MH does not allow for the nuanced gradations of problem severity available from instruments such as the Addiction Severity Index^4^ or the Global Assessment of Individual Needs.^5^ Also relevant is that there is a separate suite of tools for assessment and treatment planning, offering better coverage of the above issues, mandated for use across Ontario’s addiction treatment system.^6^

Fifth, Perlman et al^1^ note that we miscalculated the scores of the Positive Symptom Scale (PSS). From our review of archived documentation, it appears that the PSS was scored out of 8 in 2007, while a newer version is scored out of 12. The data used in our study corresponded to the early years of RAI-MH implementation, which may explain this discrepancy. At any rate, scale scores are calculated automatically by the electronic assessment platform, reducing the possibility of investigator error.

We would like to close by acknowledging that findings generated from studies conducted in other settings, on other patient populations, at other points in time, and (or) using other methods could indeed differ from our findings. There is no reason to expect anything different. Our work was a case study of the implementation of a mental health assessment platform at a single, large institution. It is not surprising to us that the impressions and feedback of clinical staff obtained during the development of the RAI-MH would differ from those of clinical staff charged with coding the instrument 3 years following its provincial mandate. We maintain that these perspectives, used in conjunction with data audits and reviews, are useful in guiding the refinement of assessment procedures in continually evolving clinical settings. We continue to believe that the best course is to refrain from blaming the clinical staff or the instrument for poor performance, and to collate the evidence generated from evaluation and research of different kinds to optimize care planning and assessment procedures. We conducted our evaluation of the RAI-MH assessment system in this spirit.

## References

[1] Perlman C, Martin L, Hirdes J (2014). Utilizing routinely collected clinical assessments in mental health services: the RAI-MH. Can J Psychiatry.

[2] Urbanoski KA, Mulsant BH, Willett P (2012). Real-world evaluation of the Resident Assessment Instrument–Mental Health assessment system. Can J Psychiatry.

[3] Hilton TF (2011). The promise of PROMIS(R) for addiction. Drug Alcohol Depend.

[4] McLellan AT, Kushner H, Metzger D (1992). The fifth edition of the Addiction Severity Index. J Subst Abuse Treat.

[5] Dennis ML, White M, Titus JC (2006). Global Appraisal of Individual Needs: administration guide for the GAIN and related measures (version 5).

[6] Centre for Addiction and Mental Health (CAMH) Knowledge Exchange (2009). Admission and Discharge Criteria and Assessment Tools (ADAT) [Internet].
